# New photostabilizers for polystyrene based on 2,3-dihydro-(5-mercapto-1,3,4-oxadiazol-2-yl)-phenyl-2-(substituted)-1,3,4-oxazepine- 4,7-dione compounds

**DOI:** 10.1186/2193-1801-2-104

**Published:** 2013-03-12

**Authors:** Emad Yousif, Ayad Hameed, Nadia Salih, Jumat Salimon, Bashar Mudhaffar Abdullah

**Affiliations:** 1 Department of Chemistry, College of Science, AL-Nahrain University, Baghdad, Iraq; 2 Department of Chemistry, College of Science, Tikrit University, Salah Al-deen, Iraq; 3 School of Chemical Sciences and Food Technology, Faculty of Science and Technology, Universiti Kebangsaan Malaysia, Bangi, Selangor, 43600 Malaysia

**Keywords:** Photochemistry, PS, UV–vis Spectroscopy, Photostabilizer, UV Absorber, 1,3-Oxazepine, 1,3,4-oxadiazole

## Abstract

The photostabilization of polystyrene (PS) films by 2,3-dihydro-(5-mercapto-1,3,4-oxadiazol-2-yl)-phenyl-2-(substituted)-1,3,4-oxazepine-4,7-dione compounds was investigated. PS films containing concentration of complexes 0.5% by weight were produced by the casting method from chloroform as a solvent. The photostabilization activities of these compounds were determined by monitoring the carbonyl and hydroxyl indices with irradiation time. The changes in viscosity average molecular weight of PS with irradiation time were also tracked (using benzene as a solvent). The quantum yield of the chain scission (Φ_cs_) of these complexes in PS films was evaluated and found to range between 3.31 × 10^-6^ and 7.89 × 10^-6^. Results obtained showed that the rate of photostabilization of PS in the presence of the additive follows the trend (I > II > III > IV). According to the experimental results obtained, several mechanisms were suggested depending on the structure of the additive like UV absorption, peroxide decomposer and radical scavenger.

## Background

Polystyrene is one of the important commercial polymers, widely used in various industrial fields. One of the important uses of PS is in the manufacture of cover signals lamp of some automobiles. PS is subjected to the irradiation of sunlight on outdoor exposure (Safy & El-Laithy, 1994).

Many polymers undergo thermal oxidative degradation during processing. Over longer periods at ambient temperature polymers also deteriorate in the solid state through autooxidation and photooxidation. In outdoor applications where the materials are exposed to UV solar radiation, the energy of this radiation is sufficient to initiate photochemical reaction leading to degradation. Plastics are commonly protected against such deterioration by the addition of antioxidants, light and heat stabilizers (Yousif et al. 2010).

There is a great interest at present in the photo-oxidative degradation of polymeric materials because macromolecules have increasingly widespread commercial applications. Synthetic, semisynthetic and natural polymers undergo degradation when exposed to the natural (Grassie & Scott, 1985).

All commercial organic polymers degrade in air when exposed to sunlight as the energy of sunlight is sufficient to cause the breakdown of polymeric C-C bonds as a consequence of degradation. The resulting smaller fragments do not contribute effectively to the mechanical properties and the polymeric article becomes brittle. Thus the life of thermoplastics for outdoor applications becomes limited due to weathering (Andrady et al. 1998).

Almost all synthetic polymers require stabilization against adverse environmental effects. It is necessary to find a means to reduce or prevent damage induced by environmental components such as heat, light or oxygen. This can be achieved through addition of special chemicals, light or UV stabilizers, that are selected to be compatible with the resin and the specific application considered. The photostabilization of polymers may be achieved in many ways. The following stabilizing systems have been developed, which depend on the action of stabilizer. a) Light screeners. b) U.V. absorbers, c) Excited state quenchers, d) Peroxide decomposers and e) Free radical scavengers, of these it is generally believed that types c), d) and e) are the most effective.

There has been no attempt to investigate the photostabilization of PS films using 1,3-oxazepine compounds containing 1,3,4-oxadiazole units. The design of 1,3-oxazepine compounds and their use as photostabilizing agents for polystyrene are reported herein.

## Results and discussion

2,3-dihydro-(5-mercapto-1,3,4-oxadiazol-2-yl)-phenyl-2-(substituted)-1,3,4-oxazepine- 4,7-dione compounds have been used as additives for the photo stabilization of PS films. To assess the effectiveness of these additives for the photostabilization of PS films changes in the infrared spectra of these materialswere monitored as a function of irradiation time at 313 nm. This irradiation altered the structure of the polymer as noted by distinct changes in the spectra. Most notable was the appearance of absorption bands characteristic of carbonyl (1720 cm^-1^) and hydroxyl groups (3450 cm^-1^). (Andrady & Searle, 1989).

**Scheme 1:**
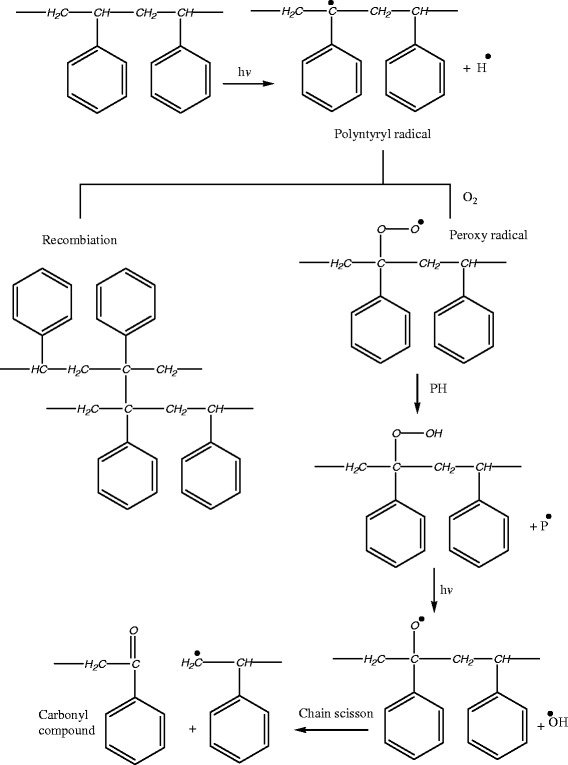
**Photooxidation reaction Scheme of PS.**

**Figure 1:**
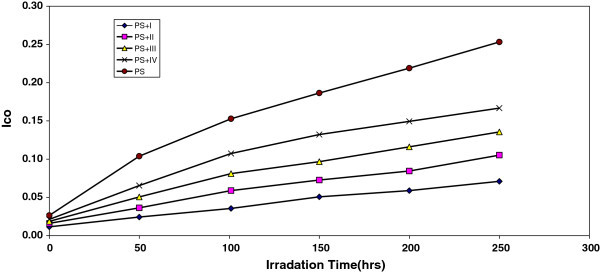
**The relationship between the carbonyl index and irradiation time for PS films (40 μm thickness) containing different additives.** Concentration of additives is fixed at 0.5% by weight.

**Figure 2:**
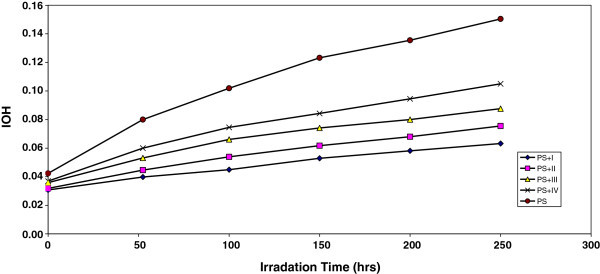
**The relationship between the hydroxyl index and irradiation time for PS films (40 μm thickness).** Containing different additives concentration of additives are fixed at 0.5% by weight.

The absorption of the carbonyl and hydroxyl groups was used to follow the extent of polymer degradation during irradiation. This absorption was calculated as carbonyl index (I_co_) and hydroxyl index (I_OH_). It is reasonable to assume that the growth of carbonyl index is a measure to the extent of degradation. A probable mechanism illustrating the experimental finding can be represented as follows (Safy & El-Laithy, 1994) (Scheme 1):

However, in Figure 1, the I_co_ of IV, III, II, and I showed lower growth rate with irradiation time with respect to the PS control film without additives. Since the growth of carbonyl index with irradiation time is lower than PS control, as seen in Figure 1, it is suitable to conclude that these additives might be considered as photostabilizers of PS polymer. Efficient photostabilizer shows a longer induction period. Therefore, the I considered as the most active photostabilizer, followed by II, III, and VI which is the least active. Just like carbonyl, hydroxyl compounds are also produced during photodegradation of PS. Therefore, hydroxyl index could also be monitored with irradiation time in the presence and absence of these additives. Results are shown in Figure 2.

### I) Variation of Ps molecular weight during photolysis in the presence of by 1,3-oxazepine compounds

**Figure 3:**
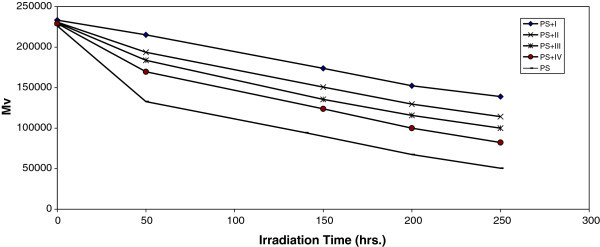
**Changes in the viscosity-average molecular weight (Mv) during irradiation of PS films (40 μm) (blank) and with 0.5 wt% of additives.**

Analysis of the relative changes in viscosity average molecular weight **,** has been shown to provide a versatile test for random chain scission. Figure 3 shows the plot of  versus irradiation time for PS film with and without 0.5% (wt/wt) of the selected additives, with absorbed light intensity of 1.052 × 10^-8^ ein. dm^-3^. s^-1^.  is measured using equation (3) with benzene as a solvent at 25°C.

It is worth mentioning that traces of the films with additives are not soluble in chloroform indicating that cross-linking or branching in the PS chain does occur during the course of photolysis (Mori et al. 1977). For better support of this view, the number of average chain scission (average number cut per single chain) (S) (Shyichuk & White 2000) was calculated using the equation (8):1

**Figure 4:**
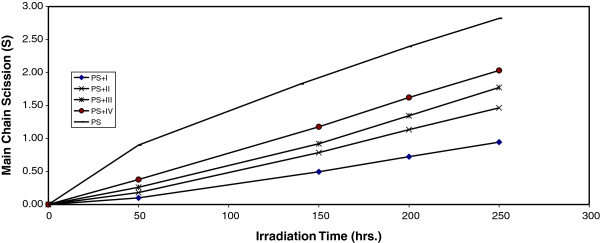
**Changes in the main chain scission (S) during irradiation of PS films (40 μm) (blank) and with 0.5 wt% of additives.**

Where  and  are viscosity average molecular weight at initial (0) and t irradiation time respectively. The plot of S versus time is shown in Figure 4. The curve indicates an increase in the degree of branching such as that might arise from cross-linking occurrence. It is observed that insoluble material was formed during irradiation which provided an additional evidences to the idea that cross-linking does occur.

For randomly distributed weak bond links, which break rapidly in the initial stages of photodegradation, the degree of deterioration α is given as:2

Where m is the initial molecular weight.

**Figure 5:**
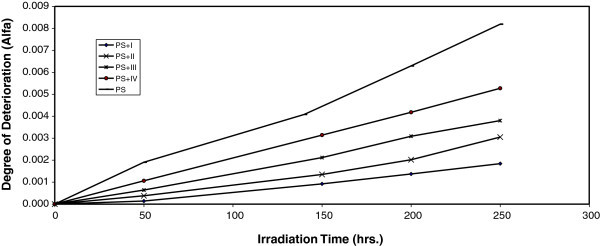
**Changes in the degree of deterioration (α) during irradiation of PS films (40 μm) (blank) and with 0.5 wt% of additives.**

The plot of α as a function of irradiation time is shown in Figure 5.

The values of α of the irradiated samples are higher when additives are absent and lower in the presence of additives compared to the corresponding values of the additive free PS. In the initial stages of photodegradation of PS, the value of α increases with time, these indicators indicates a random breaking of bonds in the polymer chain.

Another way of degradation reaction characterization is the measurement of the quantum yield of the chain scission (Φ_cs_). The quantum yield for chain scission was calculated for PS films with and without 0.5% (wt/wt) of additive mentioned above using relation (5). The Φ_cs_ values for complexes are tabulated in Table 1.

**Table 1:** **Quantum yield (Φcs) for the chain scission for PS films (40 μm) thickness with and without 0.5 (wt/wt) additive after 250 hrs irradiaton time**

Additive 0.5%(wt/wt)	Quantum yield of main chain scission (Φ_cs_)
PS + I	3.31E-06
PS + II	4.20E-06
PS + III	5.78E-06
PS + IV	6.25E-06
PS(blank)	9.67E-05

The Φ_cs_ values for PS films in the presence of additive are less than that of additive free PS (blank), which increase in the order:I, II, III, and IV

The explanation for low values of Φ_cs_ is that in macromolecule of PS, the energy is absorbed at one site, and then the electronic excitation is distributed over many bonds so that the probability of a single bond breaking is small, or the absorbed energy is dissipated by non reactive processes.

### II) Suggested mechanisms of photostabilization of Ps by 1,3-oxazepine compounds

**Scheme 2:**
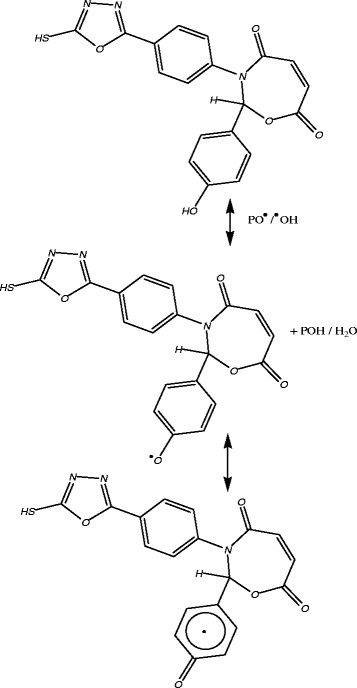
**The suggested mechanism of photostabilization of PS by compounds [1] as radical scavenger.**

**Scheme 3:**
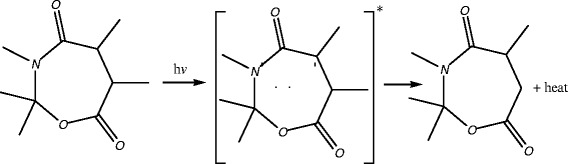
**Suggested mechanism of photostabilization of 1,3-Oxazepine ring as UV absorber.**

**Scheme 4:**
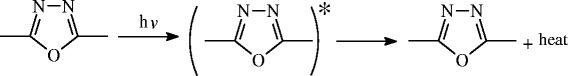
**Suggested mechanism of photostabilization of 1,3,4- oxadiazole ring as UV absorber.**

1,3-Oxazepine compounds stabilize PS by different mechanisms such as UV absorber, screener or by radical scavenger. These stabilizers provide very good long-term stability and are usually referred to these mechanisms. The most probable mechanisms involved in a photostabilization is the hydroxyl group of the additive might acts as radical scavenger for photostabilization process. Therefore this Schiff bases, besides acting as UV absorber they may also act as radical scavenger additives^19^, Scheme 2. The rings of oxazepine play a role in the mechanism of the stabilizer process by acting as UV absorber. The UV light absorption by these additives containing 1,3-Oxazepine dissipates the UV energy to harmless heat energy Scheme 3. The ring of 1,3,4-oxadiazole in this compound plays a role in the mechanism of the stabilizer process by acting as UV absorber. The UV light absorption by these additives containing 1,3,4- oxadiazole dissipates the UV energy to harmless heat energy Scheme 4, which support these compounds as photostabilizer.

## Experimental

### Materials

**Scheme 5:**
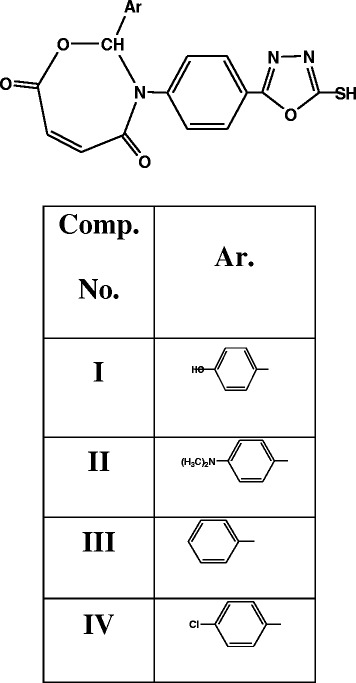
**(2,3-dihydro-(5-mercapto-1,3,4-oxadiazol-177 2-yl)-phenyl-2-(substituted)-1,3,4-oxazepine- 4,7-dione compounds)**

The following 2,3-dihydro-(5-mercapto-1,3,4-oxadiazol-2-yl)-phenyl-2-(substituted)-1,3,4-oxazepine- 4,7-dione compounds were all prepared by the method previously described by (Hameed 2012) (Scheme 5).

### Experimental techniques

#### I) Films preparation

Commercial polystyrene supplied by Petkim Company (Turkey) was re-precipitated from chloroform solution by alcohol several times and finally dried under vacuum at room temperature for 24 hours. Fixed concentrations of polystyrene solution (5 g/100 ml) in chloroform were used to prepare polymer films with 40 μm thickness (measured by a micrometer type 2610 A, Germany). The films were prepared by evaporation technique at room temperature for 24 hours (Sastre et al. 1990). To remove the possible residual chloroform solvent, film samples were further dried at room temperature for three hours under reduced pressure. The films were fixed on stands specially used for irradiation. The stand is provided with an aluminum plate (0.6 mm in thickness) supplied by Q-panel company.

#### II) Irradiation experiments

**Accelerated testing technique** Accelerated weatherometer Q.U.V. tester (Q. panel, company, USA), was used for irradiation of PS films. The accelerated weathering tester contains stainless steel plate, which has two holes in the front side and a third one behind. Each side contains a lamp (type Fluorescent Ultraviolet Lights) 40 Watt each. These lamps are of the type UV-B 313 giving spectrum range between 290–360 nm with a maximum wavelength 313 nm. The polymer film samples were vertically fixed parallel to the lamps to make sure that the UV incident radiation is perpendicular to the samples. The irradiated samples were rotated from time to time to ensure that the intensity of light incident on all samples is the same.

#### III) Photodegradation measuring methods

**A. Measuring the photodegradation rate of polymer films using infrared spectrophotometery** The degree of photodegradation of polymer film samples was followed by monitoring FTIR spectra in the range 4000–400 cm^-1^ using FTIR 8300 Shimadzu Spectrophotometer. The position of carbonyl absorption is specified at 1720 cm^-1^ and the hydroxyl group at 3450 cm^-1^ (Rabek & Ranby, 1975). The progress of photodegradation during different irradiation times was followed by observing the changes in carbonyl and hydroxyl peaks. Then carbonyl and hydroxyl indices were calculated by comparison of the FTIR absorption peak at 1720 and 3450 cm^-1^ with reference peak at 1450 cm^-1^_,_ respectively. This method is called band index method which includes (Rabek & Ranby, 1975):3

As = Absorbance of peak under study

Ar = Absorbance of reference peak

Is = Index of group under study

Actual absorbance, the difference between the absorbance of top peak and base line (A Top Peak – A Base Line) is calculated using the Base Line method (Rabek & Ranby, 1975).

B. Determination of average molecular weight  using viscometry method.

The viscosity property was used to determine the average molecular weight of polymer, using the Mark- Houwink relation (Mark, 2007).4

[η] = the intrinsic viscosity

K, α are constants depending upon the polymer-solvent system at a particular temperature.

The intrinsic viscosity of a polymer solution was measured with an Ostwald U-tube viscometer. Solutions were made by dissolving the polymer in a solvent (g/100 ml) and the flow times of polymer solution and pure solvent are t and t_0_ respectively. Specific viscosity (η_sp_) was calculated as follows:5

η_re_ = Relative viscosity.6

The single – point measurements were converted to intrinsic viscosities by the relation 2.7

C = Concentration of polymer solution (g/100 ml).

By applying equation 5, the molecular weight of degraded and undergirded polymer can be calculated. Molecular weights of PS with and without additives were calculated from intrinsic viscosities measured in benzene solution using the following equation;8

The quantum yield of main chain scission (ф_cs_) (Nakajima et al. 1990) was calculated from viscosity measurement using the following relation (9).9

Where: C = concentration; A = Avogadro’s number;  = the initial viscosity–average molecular weight; [η_o_] = Intrinsic viscosity of PS before irradiation; I_o_ = Incident intensity and t = Irradiation time in second.

## Conclusions

In the work described in this paper, the photostabilization of PS films using 2,3-dihydro-(5-mercapto-1,3,4-oxadiazol-2-yl)-phenyl-2-(substituted)-1,3,4-oxazepine4,7-dione compounds were studied. These additives behave successfully as photostabilizer for PS films. These additives stabilize the PS films through UV absorption or screening, peroxide decomposer and radical scavenger mechanisms. The compound I was found to be the most efficient in photostabilization process according to the photostability and mechanisms mentioned above. These mechanisms support the idea of using 1,3-oxazepine compounds
